# Training creative cognition: adolescence as a flexible period for improving creativity

**DOI:** 10.3389/fnhum.2014.00827

**Published:** 2014-10-29

**Authors:** Claire E. Stevenson, Sietske W. Kleibeuker, Carsten K. W. de Dreu, Eveline A. Crone

**Affiliations:** ^1^Brain and Development Lab, Developmental and Educational Psychology Department, Institute of Psychology, Leiden UniversityLeiden, Netherlands; ^2^Methodology and Statistics Department, Institute of Psychology, Leiden UniversityLeiden, Netherlands; ^3^Department of Psychology, University of AmsterdamAmsterdam, Netherlands

**Keywords:** divergent thinking, creative ideation, cognitive training, alternative uses task, adolescence

## Abstract

Creativity commonly refers to the ability to generate ideas, solutions, or insights that are novel yet feasible. The ability to generate creative ideas appears to develop and change from childhood to adulthood. Prior research, although inconsistent, generally indicates that adults perform better than adolescents on the alternative uses task (AUT), a commonly used index of creative ideation. The focus of this study was whether performance could be improved by practicing alternative uses generation. We examined the effectiveness of creative ideation training in adolescents (13–16 years, *N* = 71) and adults (23–30 years, *N* = 61). Participants followed one of three types of training, each comprising eight 20-min practice sessions within 2 week time: (1) alternative uses generation (experimental condition: creative ideation); (2) object characteristic generation (control condition: general ideation); (3) rule-switching (control condition: rule-switching). Progression in fluency, flexibility, originality of creative ideation was compared between age-groups and training conditions. Participants improved in creative ideation and cognitive flexibility, but not in general ideation. Participants in all three training conditions became better in fluency and originality on the AUT. With regard to originality, adolescents benefitted more from training than adults, although this was not specific for the creative ideation training condition. These results are interpreted in relation to (a) the different underlying processes targeted in the three conditions and (b) developmental differences in brain plasticity with increased sensitivity to training in adolescents. In sum, the results show that improvement can be made in creative ideation and supports the hypothesis that adolescence is a developmental stage of increased flexibility optimized for learning and explorative behavior.

## Introduction

Creativity is considered one of humans most complex as well as important behaviors. Its effects are evident and widespread, recognized in domains ranging from daily life problem solving to science and the arts. Creativity commonly refers to the ability to generate ideas, solutions, or insights that are novel yet feasible (e.g., Mumford, 2003). Within the *creative cognition* framework (e.g., Ward et al., 1999), creative capacity is considered inherent to normative human cognitive functioning, rather than an innate talent available to only a select few. The ability to create and use new mental categories to organize our experiences, and the ability to mentally manipulate objects are some examples of creativity that support the creative cognition approach (Ward et al., 1999). The creative cognition framework and more recent dual-processing models of creativity emphasize the dependence of creative thinking on fundamental cognitive processes such as working memory and executive control (Nijstad et al., 2010; Sowden et al., 2014). As such, individual differences in creativity can be understood in terms of variations in the efficiency of such cognitive processes (e.g., Ward et al., 1999). Furthermore, the development and malleability of the underlying mental operations used in creative problem solving processes (e.g., Klingberg, 2010; Jolles et al., 2011; Karbach and Schubert, 2013) imply that creativity develops with training and age. Indeed, numerous studies have demonstrated the effectiveness of interventions geared toward improving creativity—training in divergent thinking particularly influences performance gains in terms of originality, and to a lesser extent fluency and flexibility (e.g., Scott et al., 2004). Moreover, studies show that practice with creative ideation is highly effective in both adults (Glover, 1980; Bott et al., 2014; Kienitz et al., 2014), and children (Torrance, 1972; Cliatt et al., 1980).

In this study we examine the possibility that creative ideation develops from adolescence to adulthood, and can be trained with relatively simple interventions. Adolescence is a phase of development characterized by flexible adaption to a rapidly changing social landscape marked by changes from dependency to autonomy and individuality (Crone and Dahl, 2012). It forms a crucial phase for the development of cognitive abilities assumed to be related to creative cognition such as working memory and cognitive control (e.g., Diamond et al., 2002; Bunge and Wright, 2007; Huizinga and van der Molen, 2007; Crone and Dahl, 2012). Yet, relatively little is known about whether and how malleable divergent thinking is in adolescence. Training in other higher cognitive skills such as working memory (Klingberg, 2010; Jolles et al., 2012), executive control (Karbach and Kray, 2009; Zinke et al., 2012), relational reasoning (Dumontheil et al., 2010), and algebraic equation solving (Qin et al., 2004) emphasize the plasticity of the adolescent brain. In this study we test this hypothesis with regard to the development of creative ideation skills.

Creative ideation can be tracked with the Alternative Uses Task (AUT, Guilford, 1967; Kim, 2008), in which participants generate alternative uses for a common object (e.g., a brick; with alternative, original uses such as “making music” or “Geisha pillow”). These ideas are typically coded for three core components of creative ideation: originality or uniqueness (less frequent is considered more original), flexibility (more semantic categories implies more flexible), and creative fluency (more ideas translates to greater fluency). Especially originality improves with age (e.g., Runco and Bahleda, 1986; Urban, 1991; Lau and Cheung, 2010; Kleibeuker et al., 2013a)—although performance slumps at different stages in adolescence may occur (Lau and Cheung, 2010). Studies comparing adolescents and adults on the AUT often reveal advantages for adults. For example, Kleibeuker et al. (2013c) found that adults' AUT solutions were more unique than those of 12–13 and 15–16 year olds.

Results with regard to fluency and flexibility are more mixed. In some studies no differences were found between adolescents and adults (Wu et al., 2005; Kleibeuker et al., 2013c). In contrast, Kleibeuker et al. (2013b) found that late adolescents of 15–17 years had lower fluency and flexibility scores, but not originality scores, than adults on the AUT. Furthermore, Jaquish and Ripple (1981) found that adolescents obtained higher fluency and flexibility scores, but not originality scores, compared to children. On the whole, in the verbal divergent thinking domain applied in this study, adolescents generally provide less original solutions and, especially in late adolescence, show less fluency and flexibility than adults.

The present study aimed to extend investigations into the development of creative ideation by examining the progression of adults and adolescents within a simple training paradigm. The main question was whether creative ideation in adolescents is limited by maturational constraints or that exposure to divergent thinking training leads to progression in creative ideation thereby narrowing the gap in performance between adolescents and adults. To this end, participants were asked to practice generating alternative uses for everyday objects over a 2 week period. To examine the effects of training two active control groups were employed (Jolles and Crone, 2012), both trained in cognitive processes that were associated with but not directly related to creative ideation. One control group generated ordinary characteristics of everyday objects (adapted from Fink et al., 2009). This task has successfully served as a general ideation control task (Fink et al., 2009, 2010; Kleibeuker et al., 2013b). The second active control group practiced in rule-switching. Here, participants were asked to quickly and accurately apply and switch between two rule sets (Huizinga et al., 2010).

Given findings from previous research, routine practice in alternative uses generation for everyday objects was expected to improve creative performance over the course of a short, but intensive training period for both adolescents and adults. Participants who practiced generating alternative uses (creativity training condition) were expected to improve more on measures of creative fluency, flexibility and originality compared to the active control group. Adults were expected to initially provide more creative solutions to the AUT than adolescents on originality, and perhaps fluency and flexibility (Kleibeuker et al., 2013a,b); however, adolescents were expected to improve more over the course of training based on the hypothesis that adolescence is a period of enhanced sensitivity to training of high-level cognitive skills compared to adults (Steinberg, 2005; Jolles and Crone, 2012).

## Materials and methods

### Participants

The sample comprised 71 adolescents (*M_age_* = 14.9, *SD* = 0.7, Range = 13.0–16.2 years, 67% females) and 61 adults (*M_age_* = 25.3, *SD* = 2.4, Range = 22.1–31.1 years, 50% females). Adolescents were recruited from local high schools (college preparation level) and adults were recruited from Leiden University and colleges in The Hague. All participants provided informed consent. In case of minors, consent was also obtained from primary caregivers. Participation was compensated with gift vouchers, money, or course credits. All procedures were approved by the Internal Review Board of Leiden University Institute of Psychology.

The data was gathered in two waves separated by 15 months. In both waves adolescents and adults were recruited and randomly assigned to one of the training conditions (creative ideation; general ideation; rule-switching). There were two drop-outs. During the pretest and posttest not all data was available for all participants on all tasks. In some cases this was due to technical errors and in other cases students were absent from a testing session. Because the data was missing at random and not due to selection bias or systematic error, the validity of the statistical tests was not affected (Schafer and Graham, 2002). The number of subjects used in statistical analyses is reported separately per task and, as recommended, and Maximum Likelihood estimation was used when appropriate.

#### General cognitive ability

Creativity is associated with verbal fluency (Gilhooly et al., 2007), fluid reasoning (Nusbaum and Silvia, 2011), and working memory (De Dreu et al., 2012). Tasks that measure these constructs were administered at pretest in order to check for any differences between training conditions. The verbal fluency test (subtest of the Groningen Intelligence Test, GIT-2, Luteijn and Barelds, 2004) was used to measure general verbal ideation ability. Fluid reasoning was measured with the Raven Advanced Progressive Matrices (APM, Raven et al., 1998). Working memory was assessed using the mental counters task (Huizinga et al., 2006). Analyses of Variance were conducted with Age (adolescent, adult) and Training Condition (creative ideation, general ideation and rule-switching) as between-subjects factors to assess any differences in performance on these three tasks. See Tables 1, 2 for descriptive statistics and *F*-test results, respectively. No age group or training condition differences were found with regard to fluid reasoning. Adults outperformed adolescents on the measures of verbal fluency and working memory; however, there were no significant effects for training condition or age-group by training condition.

**Table 1:** **Descriptive statistics of pretest and posttest measures per training condition and age group on control variables: fluid reasoning, verbal fluency, and working memory**.

	**Creative ideation training**			**General ideation training**			**Rule-switch training**		
	***N***	***M***	***SD***	***N***	***M***	***SD***	***N***	***M***	***SD***
**VERBAL FLUENCY**									
Adolescents	25	23.16	3.44	23	22.96	5.09	21	23.38	5.18
Adults	21	27.24	5.07	19	26.84	8.30	19	24.79	6.72
**RAVEN APM**									
Adolescents	23	9.26	2.01	21	9.38	3.14	20	8.45	3.58
Adults	19	9.68	1.60	18	10.11	3.32	16	10.00	1.97
**WORKING MEMORY**									
***Adolescents***									
Accuracy	19	0.88	0.08	16	0.86	0.09	18	0.84	0.19
Reaction time^*^	19	574	98	16	594	122	18	534	89
***Adults***									
Accuracy	20	0.90	0.09	19	0.91	0.09	18	0.92	0.05
Reaction time^*^	20	487	115	19	555	152	18	502	106

* *Reaction time is reported in milliseconds*.

**Table 2:** ***F*-test results for comparisons of general cognitive ability measures verbal fluency, fluid reasoning, and working memory per training condition and age group**.

	***F***	***df***	***p***	**η^2^_*p*_**
**VERBAL FLUENCY**				
Age	9.54	1, 122	<0.01	0.07
Condition	0.43	2, 122	0.65	0.01
Age × Condition	0.70	2, 122	0.50	0.01
**RAVEN APM**				
Age	3.17	1, 111	0.08	0.03
Condition	0.34	2, 111	0.71	0.01
Age × Condition	0.43	2, 111	0.65	0.01
**WORKING MEMORY**				
***Accuracy***				
Age	5.95	1, 104	0.02	0.05
Condition	0.05	2, 104	0.95	0.00
Age × Condition	0.68	2, 104	0.51	0.01
***Reaction time***				
Age	5.55	1, 104	0.02	0.05
Condition	2.34	2, 104	0.10	0.04
Age × Condition	0.65	2, 104	0.53	0.01

*VF, verbal fluency; WM, working memory. Reaction time is reported in milliseconds*.

### Design and procedure

A pretest-training-posttest design with three training conditions (creative ideation, general ideation, rule-switching) and two age groups (adolescents, adults) was employed, yielding a 2 (pre/post) × 2 (Age group) × 3 (training) factorial with the second and third factor between-subjects.

During the pretest session, all participants were administered two tasks measuring creative ideation, the AUT “Tin Can” task and the Alternative Uses part of a combined Alternative Uses/Ordinary Characteristics task (AU/OC task). General ideation was assessed using the Ordinary Characteristics part of the AU/OC task. A rule-switching task was also administered. In addition, verbal fluency, working memory, and fluid reasoning were assessed in order to ascertain whether the three training × two age groups did not differ on these control variables prior to training.

In the 2 weeks following the pretest session, participants followed an online training during their free time at home or at school. Participants were randomly assigned to one of three different trainings: creative ideation, general ideation, or rule-switching. Participants were asked to train eight times with a minimum of 1 day and a maximum of 2 days between training sessions and received an email or text message when needed to prompt them to train on time.

The posttest session comprised of the same tasks as the pretest and was administered 1 or 2 days following the last training session.

### Instruments

#### Creative ideation

***Alternative Uses Test: pretest and posttest***. A computerized 4-min version of the Alternative Uses Test (AUT; Guilford, 1950, 1967) was administered to measure creative ideation. Participants were given the name of an object and asked to generate as many alternative uses for the object as possible within a 4 min period (e.g., Friedman and Förster, 2001). At pretest the object was “Tin Can” and at posttest the object was “Brick.” Participants were instructed to type in their solutions one at the time. After submitting the solution the text was no longer shown on the screen. From the generated ideas, we derived indices of fluency, flexibility, and originality after removing erroneous solutions (e.g., empty solutions, random strings such as “asdfjk;” and non-sense solutions such as “blah”). O*riginality* was rated on a 5-point scale (from 1 = “not original” to 5 = “highly original”) by trained raters according to a pre-specified scheme (Rietzschel et al., 2006; De Dreu et al., 2008). The interrater reliability of the originality scores of this task were ICC = 0.91. *Fluency* scores were the sum of correct solutions provided. *Flexibility* was measured by the number of solution-categories per participant after trained raters assigned each solution to a set of predefined solution-categories (e.g., building aspect; load; toy; Rietzschel et al., 2006; De Dreu et al., 2008). *Unicity* provides an indication of how unique a particular solution was and was scored as the number of persons who provided the same solution, where higher scores indicate less unique solutions.

***Combined Alternative Uses/Ordinary Characteristics Task: pretest and posttest***. In the combined Alternative Uses (AU) and Ordinary Characteristics (OC) task the participant was presented with an object and requested to list object properties according to the rules of the task. During AU trials participants were asked to name as many novel uses of a common object as possible (e.g., “umbrella,” example answer: “baseball bat”). During OC trials as many typical characteristics of a common object (e.g., “shoe,” example answer: “fits on a foot”) were requested. The AU trials measures creative ideation similar to the traditional Alterative Uses Test (AUT, Guilford, 1950, 1967), but now for multiple objects within a shorter time period. The OC part of the task is described in Section General Ideation.

**Figure 1:**
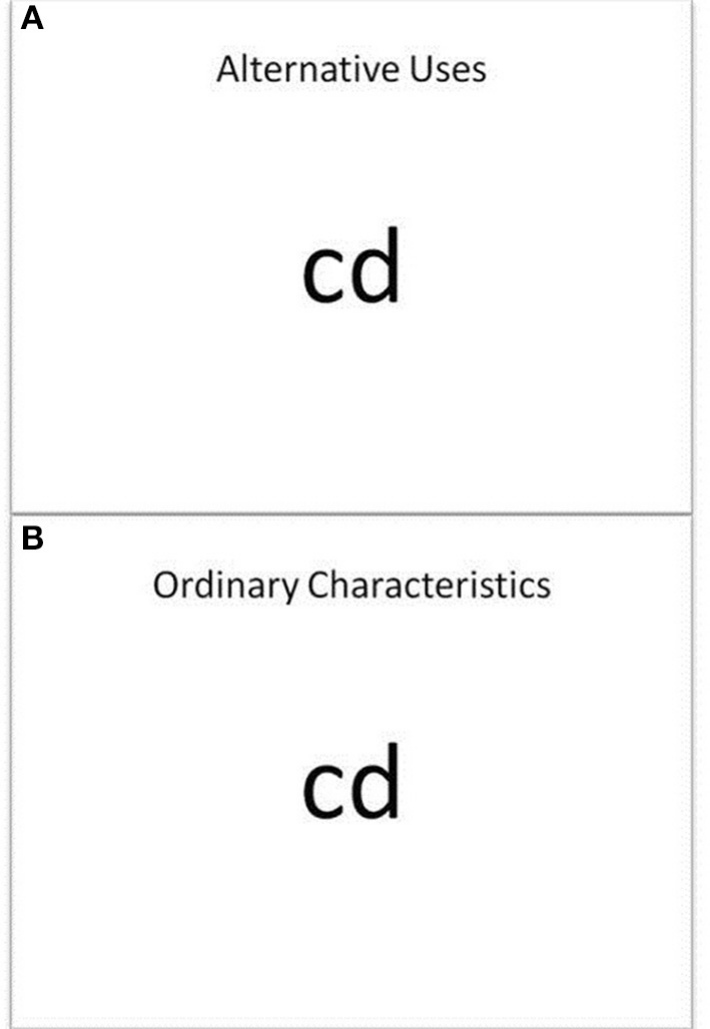
**Alternative Uses/Ordinary Characteristics task: (A) example Alternative Uses item and (B) example Ordinary Characteristics item**. Participants were asked to list as many alternative uses for *or* ordinary characteristics of an everyday object as possible.

For each trial the participant was shown an instruction screen (3 s) identifying the trial type (“alternative uses” or “ordinary characteristics”). In the next screen the target object name appeared in the middle of the screen with the instruction “alternative uses” or “ordinary characteristics” reiterated at the top of the screen (see Figure 1). The participant was given 20 s to list solutions out loud. The solutions were recorded and later transcribed. Per session 30 items (15 AU and 15 OC) were in random order, divided across two blocks (7 min each) separated by a short break. There were 60 items in total; the allocation to session (pretest, posttest) and type (AU, OC) were counterbalanced over participants and training conditions.

The AU responses were coded for creative fluency (average number of unique solutions across trials), and originality (the average rating across AU trials per stimulus). Two independent trained raters assessed originality on this measure with interrater reliability ICC = 0.73.

***Alternative Uses: training***. Participants in the AU training condition trials were administered 10 AU items during each of the eight training sessions. The items lasted 2 min each. A short break was provided halfway through the training. Each session began with a brief: “Generate as many alternative uses for each presented object.” This was followed by one screen per item with the instruction briefly reiterated at the top of the screen. The participant typed the solutions into a text box and each submitted solution was posted below on the same screen. After 2 min the next item was shown. A total of 80 stimuli were presented in random order across trials over training sessions. The total duration of training was approximately 20 min.

The AU training sessions were coded for *originality* (the average rating across trials per stimulus) and creative *fluency* (average number of unique solutions across trials within one session). *Flexibility* (the number of categories used from a set of predefined solution-categories) was also measured for the first trial per training session.

#### General ideation

***Combined Alternative Uses/Ordinary Characteristics Task: pretest and posttest***. General ideation is the second skill assessed in the combined Alternative Uses (AU) and Ordinary Characteristics (OC) task. The OC task was based on Fink et al. (2009) and served as a general control for the creative ideation training, appealing to memory retrieval processes. For each OC trial the participant was shown an instruction screen (3 s) identifying the trial type (“ordinary characteristics”). In the next screen the target object name appeared in the middle of the screen with the instruction “ordinary characteristics” reiterated at the top of the screen (see Figure 1). The participant was given 20 s to list solutions out loud. The solutions were recorded and later transcribed. Per session 15 OC trials (and 15 AU trials) were presented in random order. There were 30 OC items in total across pretest and posttest; the allocation to session (pretest, posttest) and type (AU, OC) was counterbalanced over participants and training conditions.

***Ordinary Characteristics: training***. Participants in the general ideation condition were asked to solve 10 OC items lasting 2 min each, with a short break halfway, during each of the eight training sessions. Each session began with a brief instruction “List as many ordinary characteristics as possible for the object on the screen.” This was followed by one screen per item with the instruction briefly reiterated at the top of the screen. The participant typed solutions into a text box and each submitted solution was posted below on the same screen. After 2 min the next item was shown. A total of 80 stimuli were presented in random order across trials over sessions. The total duration of the general ideation training was approximately 20 min. The OC responses were coded for fluency, i.e., the average number of correct solutions across all OC trials within the session.

#### Rule-switching

**Figure 2:**
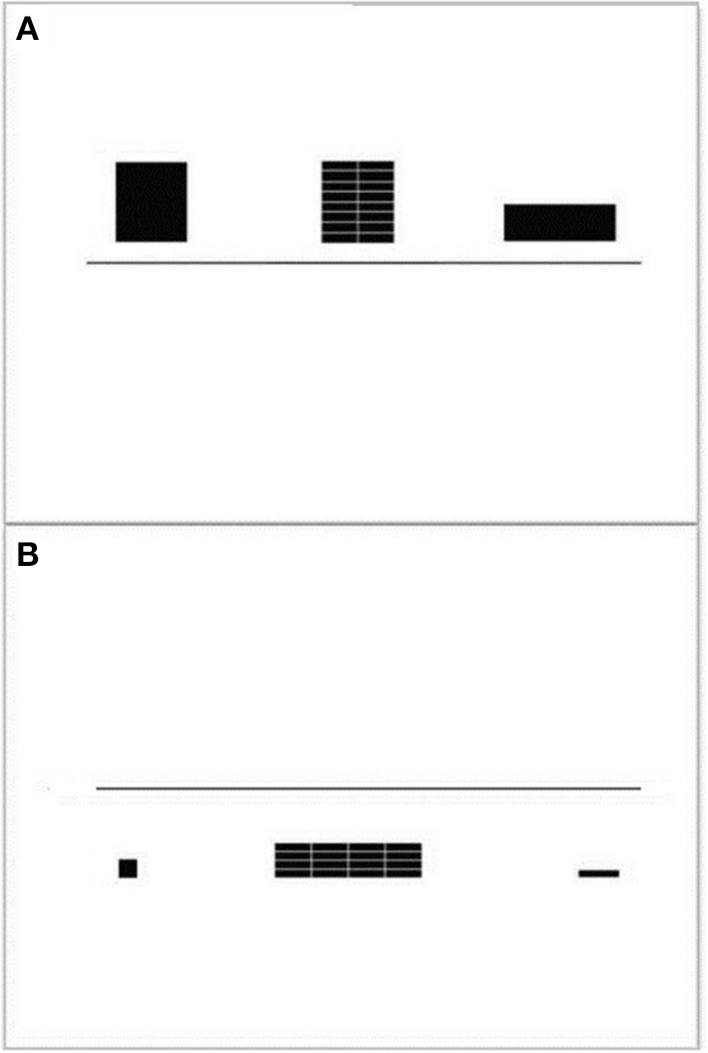
**Rule-switching task example items: (A) global rule and (B) local rule**. The participant was cued to apply the global rule to the figure in the middle when the two side figures were large. The local rule was applied if the side figures were small. Switch blocks involved applying both rules in random order. In both figures above the correct solution is on the left-hand side, thus the participant would press the left button.

Rule-switching was measured and trained with the global/local rule-switch (RS) task (Huizinga et al., 2010). Participants were shown a rule comprising of two objects: (1) a large square and a rectangle (global rule) or (2) a small square and a small rectangle (local rule). Next the stimulus, a large square or rectangle composed of smaller squares or rectangles (2 × 2 possible stimuli), was presented in between the two rule objects. During this time the participant was asked to indicate which rule the stimulus belonged to. The decision rule was based on the size of the square and rectangle on either side of the target. If the side figures were large the “global” rule was to be applied—i.e., indicate the stimulus as a whole was a large square or rectangle. If the side figures were small then the “local” rule was required—i.e., indicate whether the stimulus was composed of small squares or rectangles. See Figure 2 for an example. During the first and second blocks of this task decisions were based on only one rule (“global” or “local”). During the remaining blocks the two rules were mixed and the participant had to switch between the rules. The switching costs for accuracy and reaction time computed using the ration between rule repeat trials and trials directly following a rule switch.

***Rule-switching: pretest and posttest***. Four blocks of 50 trials were administered. The task lasted approximately 10 min.

***Rule-switching: training***. Four blocks of 80 trials each were administered. The total duration of a RS training session was approximately 20 min, including a short break between blocks two and three.

## Results

### Descriptive statistics

Descriptive statistics for all pretest and posttest measures per age group (adolescent, adult) and training condition (creative ideation, general ideation, and rule-switching) are shown in Table 3. Correlations between each of the pretest and posttest measures are shown in Table 4.

**Table 3:** **Descriptive statistics of pretest and posttest measures per training condition and age group on the combined Alternative Uses/Ordinary Characteristics task (AU/OC task), the Alternative Uses test and the Rule-Switching task**.

	**Creative ideation training**			**General ideation training**			**Rule-Switch training**		
	***N***	***M***	***SD***	***N***	***M***	***SD***	***N***	***M***	***SD***
	**Pretest**								
**COMBINED AU/OC PRETEST**									
***Adolescents***									
AU originality	23	2.08	0.36	22	2.19	0.41	22	1.99	0.40
AU fluency	23	2.46	0.81	22	2.25	0.73	22	2.76	1.13
OC fluency	23	3.53	1.30	22	3.69	0.85	22	3.75	1.26
***Adults***									
AU Originality	21	2.37	0.41	19	2.25	0.28	17	2.49	0.39
AU fluency	21	2.65	0.87	19	2.83	0.99	17	2.27	0.71
OC fluency	21	4.16	1.24	19	4.63	1.49	17	4.43	0.89
**ALTERNATIVE USES PRETEST**									
***Adolescents***									
Fluency	25	11.92	5.53	23	12.83	6.55	23	12.70	6.72
Flexibility	25	6.16	2.17	23	5.91	2.17	23	6.17	2.76
Originality	25	1.68	0.35	23	1.64	0.27	23	1.69	0.34
***Adults***									
Fluency	22	11.41	3.45	20	11.65	5.71	19	12.74	5.51
Flexibility	22	6.91	1.82	20	5.70	3.08	19	6.95	2.12
Originality	22	1.73	0.37	20	1.75	0.33	19	1.67	0.29
**RULE-SWITCHING PRETEST**									
***Adolescents***									
Accuracy	17	0.00	0.09	14	0.03	0.06	14	0.02	0.04
Reaction time^*^	17	104	115	14	81	76	14	70	35
***Adults***									
Accuracy	21	0.00	0.04	19	0.00	0.08	18	0.00	0.05
Reaction time^*^	21	55	54	19	79	52	18	100	88
	**Posttest**								
**AU/OC POSTTEST**									
***Adolescents***									
AU originality	25	2.46	0.18	19	2.50	0.27	18	2.47	0.24
AU fluency	25	2.99	1.35	19	2.55	0.85	18	3.15	1.12
OC fluency	25	3.69	0.96	19	4.20	1.02	18	4.23	1.30
***Adults***									
AU Originality	21	2.61	0.22	20	2.58	0.19	17	2.62	0.23
AU fluency	21	3.07	0.93	20	2.80	0.79	17	2.49	1.28
OC fluency	21	3.96	0.98	20	4.76	1.09	17	3.99	1.10
**ALTERNATIVE USES POSTTEST**									
***Adolescents***									
Fluency	23	14.35	7.99	19	11.32	7.37	20	17.25	7.15
Flexibility	23	8.48	2.11	19	7.53	2.46	20	9.85	2.80
Originality	23	1.66	0.34	19	1.79	0.49	20	1.75	0.44
***Adults***									
Fluency	18	13.39	4.47	15	12.20	4.16	16	10.81	5.74
Flexibility	18	9.44	2.18	15	8.33	2.16	16	7.38	2.96
Originality	18	1.70	0.30	15	1.60	0.24	16	1.67	0.32
**RULE-SWITCHING POSTTEST**									
***Adolescents***									
Accuracy	18	−0.03	0.04	15	−0.01	0.04	16	−0.02	0.06
Reaction time^*^	18	89	68	15	45	80	16	30	28
***Adults***									
Accuracy	20	0.00	0.04	18	0.00	0.03	20	0.02	0.07
Reaction time^*^	20	26	34	18	53	38	20	30	30

*Both versions of the alternative uses task measure creative ideation. The ordinary characteristics task measures general ideation*.

*AU, alternative uses; OC, ordinary characteristics; RS, rule-switching*,

*The rule-switching task reports switch costs*.

* *Reaction time is reported in milliseconds*.

**Table 4:** **Correlations between the pretest and posttest measures on the Alternative Uses test (AUT), combined Alternative Uses/Ordinary Characteristics task (AU/OC task), and rule-switch task (RS)**.

	**Pretest**								**Posttest**							
	**AU/OC**			**AUT**			**Rule-Switch**		**AU/OC**			**AUT**			**Rule-Switch**	
	**AU orig**	**AU flu**	**OC flu**	**orig**	**flu**	**flex**	**acc**	**rt**	**AU orig**	**AU flu**	**OC flu**	**orig**	**flu**	**flex**	**acc**	**rt**
**PRETEST TASKS**																
***Combined AU/OC***																
AU originality	1															
AU fluency	0.09	1														
OC fluency	0.36^**^	0.49^**^	1													
***Alternative uses***																
Originality	0.11	0.17	0.06	1												
Fluency	0.19^*^	0.21^*^	0.21^*^	0.05	1											
Flexibility	0.27^**^	0.19^*^	0.28^**^	0.13	0.72^**^	1										
***Rule-switch***																
Accuracy	−0.20^*^	0.01	−0.05	0.18	−0.05	−0.05	1									
Reaction time	−0.05	−0.16	−0.11	−0.14	−0.13	−0.13	−0.04	1								
**POSTTEST TASKS**																
***Combined AU/OC***																
AU originality	0.23^*^	−0.07	0.11	0.08	0.10	0.10	−0.03	−0.03	1							
AU fluency	−0.02	0.53^**^	0.27^**^	0.03	0.25^**^	0.20^*^	−0.03	−0.05	−0.04	1						
OC fluency	−0.08	0.35^**^	0.54^**^	0.09	0.27^**^	0.27^**^	0.02	−0.05	0.09	0.42^**^	1					
***Alternative uses***																
Originality	0.00	0.03	−0.01	0.14	0.09	0.10	0.01	−0.10	0.18	0.15	0.07	1				
Fluency	−0.05	0.39^**^	0.11	0.11	0.21^*^	0.14	−0.08	−0.03	−0.04	0.61^**^	0.30^**^	0.16	1			
Flexibility	0.01	0.39^**^	0.14	0.15	0.21^*^	0.25^**^	−0.08	−0.03	−0.02	0.58^**^	0.30^**^	0.03	0.79^**^	1		
***Rule-switch***																
Accuracy	0.15	−0.22^*^	0.04	−0.29^**^	−0.07	−0.04	−0.03	0.18	0.22^*^	−0.03	−0.04	0.01	−0.12	−0.15	1	
Reaction time	0.02	−0.04	−0.09	−0.17	−0.11	−0.21^*^	0.07	0.41^**^	−0.11	−0.09	−0.16	−0.15	0.06	0.03	−0.11	1

Orig, originality; flu, fluency; flex, flexibility; acc, accuracy; rt, reaction time.

* *p < 0.05*,

** *p < 0.01*.

#### Initial comparisons

**Figure 3:**
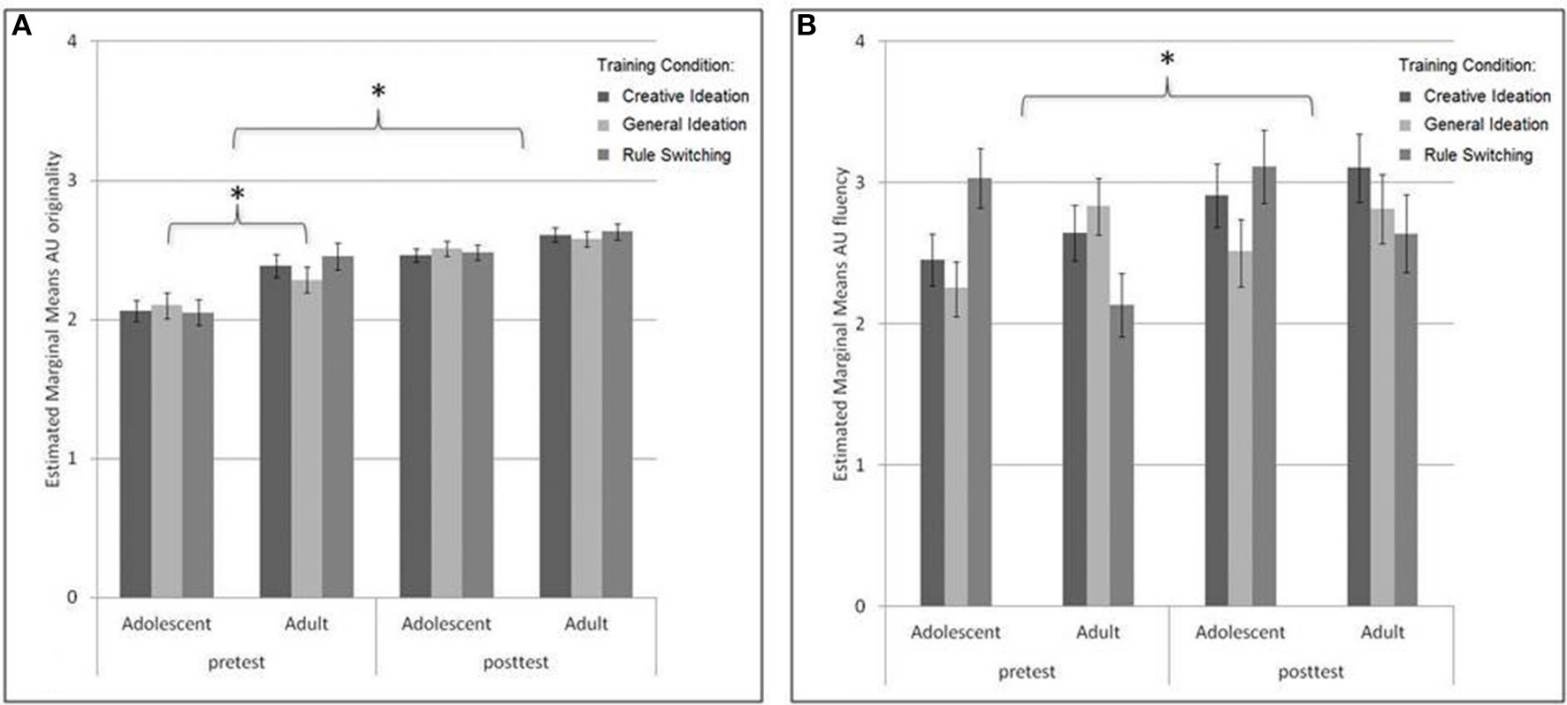
**Pretest to posttest progression for adults and adolescents on the creative ideation measure of the combined Alternative Uses/Ordinary Characteristics task: (A) originality (1 = “not original” to 5 = “highly original”) and (B) fluency (number of alternative uses listed)**. In general participants improved in AU originality from pretest to posttest. Adults had higher mean originality scores than adolescents; however, adolescents showed greater gains from pretest to posttest than adults in AU originality. Participants generally had higher mean AU fluency scores on the posttest compared to pretest; however, no age group or training condition differences were found. ^*^ *p* < 0.05.

Initial comparisons were conducted on each of the pretest tasks between the two age groups and three training conditions to examine whether differences prior to training were present. The results of the Analysis of Variance (ANOVAs) with Age and Condition as between-subjects factors are presented in Table 5. Here we see that age effects emerged on the combined AU/OC task for the measures of AU originality and OC fluency. In both cases adults obtained higher scores than adolescents. No further main effects for Age or Training Condition were found on any of the pretest creative ideation, general ideation and rule-switching tasks. Age × Training Condition effects were not present on the AUT or rule-switching tasks; however, an interaction was present on the combined AU/OC task for the AU originality and AU fluency measures. *Post-hoc* analyses with Bonferroni correction revealed that these interaction effects emerged because of Age effects in some but not all Training Conditions (see Figure 3). For AU originality Age effects, with higher scores for adults, were present for the AU and RS conditions [AU condition: *F*_(1, 42)_ = 6.12, *p* = 0.02, η^2^_*p*_ = 0.13; RS condition: *F*_(1, 37)_ = 15.40, *p* < 0.001, η^2^_*p*_ = 0.29] but not for the OC condition (*p* > 0.10). For AU fluency we found a significant Age effect for the OC condition [*F*_(1, 39)_ = 4.65, *p* = 0.04, η^2^_*p*_ = 0.11], where adults obtained higher scores, but not for the AU and RS conditions. In sum, age-group differences were present on the combined AU/OC task; however, these initial differences were accounted for in our main analyses as we applied repeated measures ANOVAs.

**Table 5:** ***F*-test results for pretest and posttest measures per training condition and age group on the combined Alternative Uses/Ordinary Characteristics task (AU/OC task), the Alternative Uses test (AUT), and the Rule-Switching task**.

	***F***	***df***	***p***	**η^2^_*p*_**
**AU/OC TASK**				
***AU originality***				
Age	**16.08**	**1, 118**	**<0.001**	**0.12**
Condition	0.14	2, 118	0.87	0.00
Age × Condition	2.79	2, 118	0.07	0.05
***AU fluency***				
Age	0.30	1, 118	0.58	0.00
Condition	0.01	2, 118	0.99	0.00
Age × Condition	**4.01**	**2, 118**	**0.02**	**0.06**
***OC fluency***				
Age	**12.75**	**1, 118**	**<0.001**	**0.10**
Condition	0.77	2, 118	0.47	0.01
Age × Condition	0.17	2, 118	0.85	0.00
**ALTERNATIVE USES TEST**				
***Originality***				
Age	0.57	1, 126	0.45	0.00
Condition	0.04	2, 126	0.96	0.00
Age × Condition	0.37	2, 126	0.69	0.01
***Fluency***				
Age	0.30	1, 126	0.58	0.00
Condition	0.38	2, 126	0.69	0.01
Age × Condition	0.12	2, 126	0.89	0.00
***Flexibility***				
Age	1.10	1, 126	0.30	0.01
Condition	1.39	2, 126	0.25	0.02
Age × Condition	0.60	2, 126	0.55	0.01
**RULE-SWITCHING TASK**				
***Switch costs accuracy***				
Age	1.47	1, 97	0.23	0.02
Condition	0.44	2, 97	0.65	0.01
Age × Condition	0.49	2, 97	0.62	0.01
***Switch costs reaction time***				
Age	0.14	1, 97	0.71	0.00
Condition	0.02	2, 97	0.98	0.00
Age × Condition	1.99	2, 97	0.14	0.04

*p < 0.05 appears in bold*.

#### Correlations

Associations between the pretest measures were in the expected directions. Firstly, AU originality measures (AUT and combined AU/OC version) were all positively correlated, although the expected association between originality on the 4-min AU Tin test and the AU/OC task was not significant. Secondly, the associations between the AU and OC fluency measures were all moderate to strong. Finally, rule-switching performance during pretest was strongly related to rule-switching performance during the RS training condition participants' first training session. In sum, the pretest correlations support the validity of our tasks.

Correlations between each of the posttest measures were generally as expected and these correlations were often stronger than during the pretest. The two AU originality measures (AUT and combined AU/OC version) were positively correlated.

Correlations between pretest and posttest measures of the same task were generally all positive but varied in strength. The correlation between the AUT originality pretest and posttest was not significant; however, as we will see in the next section this is most likely due to changes taking place in some groups but not others as will be discussed in the next Section Pretest to Posttest Change.

### Pretest to posttest change

We had two main inquiries concerning pretest to posttest change on the three training-related measures of creative ideation, general ideation and rule-switching. Our first research question concerned the effectiveness of the intervention; we expected participants within a training condition to improve more on the task they practiced than participants in the other two training conditions. Our second research question focused on differential progression from pretest to posttest between age groups; we examined whether adolescents showed greater improvement in performance than adults on all tasks.

In order to test our hypotheses concerning pretest to posttest change on the measures of creative ideation (AU tasks), general ideation (OC task) and rule-switching (RS task), repeated measures ANOVAs were conducted with Age (adolescent, adult) and Training Condition (creative ideation, general ideation, rule-switching) as between-subjects factors and Session (pretest, posttest) as within-subjects factor. Homogeneity of variance between factors was examined with Levene's test. For the AUT, equal task difficulty for the Tin Can (pretest) and Brick (posttest) versions could not be assumed. Accordingly, ANCOVAs with Age and Condition as between-subjects factors and the AUT pretest score as covariate was conducted to test our hypotheses.

#### Creative ideation

Two tasks measured creative ideation: (1) the alternative uses part of the combined Alternative Uses/Ordinary Characteristics (AU/OC) task and (2) the Alternative Uses Test. Pretest to posttest change on these two tasks was examined separately and is described in the following subsections. We hypothesized that participants trained in creative ideation would improve more in originality, fluency (number of valid creative solutions) and flexibility (ability to change categories during creative ideation) on the AU tasks than participants trained in general ideation or rule-switching.

***Alternative Uses: AU/OC task***. The alternative uses part of the combined AU/OC task comprised of measures of AU originality and AU fluency. The first set of analyses tested for training effects on AU originality scores. A main effect of Session showed that participants generally improved on the AU originality measure from pretest to posttest [*F*_(1, 98)_ = 64.02, *p* < 0.001, η^2^_*p*_ = 0.395]. A main effect of Age showed that adults obtained higher scores on the AU originality measure on the whole [*F*_(1, 98)_ = 22.53, *p* < 0.001, η^2^_*p*_ = 0.187]. A Session × Age interaction showed that adolescents progressed more from pretest to posttest on AU originality [*F*_(1, 51)_ = 61.42, *p* < 0.001] than adults [*F*_(1, 47)_ = 14.19, *p* < 0.001]: Session × Age effect: *F*_(1, 98)_ = 5.14, *p* = 0.03, η^2^_*p*_ = 0.05 (see Figure 3A). Pretest to posttest change in AU originality did not differ between training conditions [Session × Training Condition effect: *F*_(2, 98)_ = 0.13, *p* = 0.88, η^2^_*p*_ = 0.00]; Session × Age × Training Condition effect: *F*_(2, 98)_ = 0.23, *p* = 0.79, η^2^_*p*_ = 0.01.

The same analyses for AU fluency showed that in general, participants improved in AU fluency from pretest to posttest [Session effect: *F*_(1, 102)_ = 8.91, *p* < 0.01 η^2^_*p*_ = 0.09]. No significant differences in AU fluency progression were observed for Age [Age effect: *F*_(1, 98)_ = 0.01, *p* = 0.91, η^2^_*p*_ = 0.00 or Session × Age effect: *F*_(1, 102)_ = 0.10, *p* = 0.76, η^2^_*p*_ = 0.00; see Figure 3B], Condition [Session × Condition effect: *F*_(2, 102)_ = 0.90, *p* = 0.41, η^2^_*p*_ = 0.02] or Age × Condition [Session × Age × Condition effect: *F*_(2, 102)_ = 1.20, *p* = 0.31, η^2^_*p*_ = 0.02].

**Figure 4:**
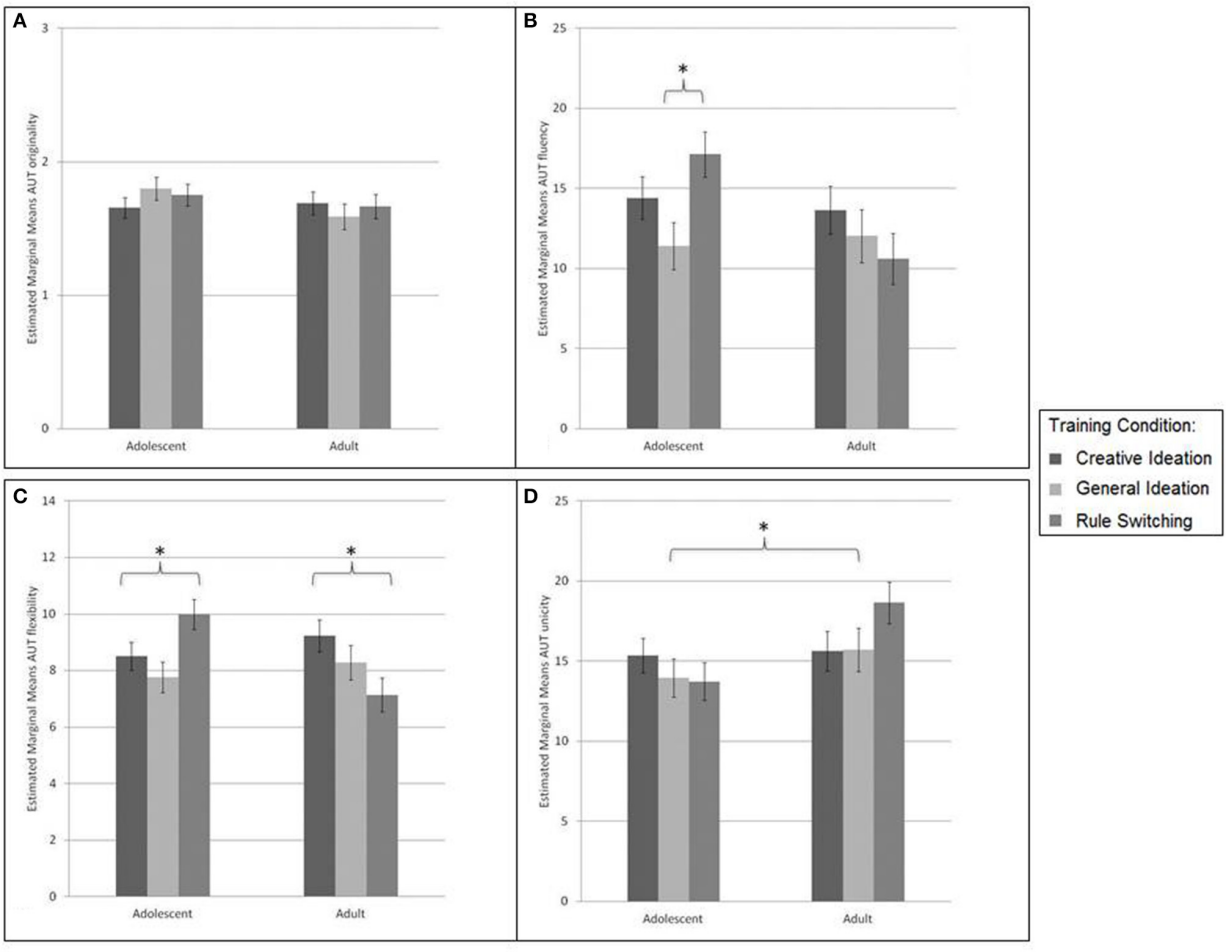
**Alternative Uses “brick” posttest performance for adults and adolescents per training condition on measures: (A) originality (1 = “not original” to 5 = “highly original”), (B) fluency (number of solutions), (C) flexibility (number of categories used in solutions), and (D) unicity (inverse of uniqueness, i.e., mean frequency of provided solution in dataset)**. No differences were found in originality between age groups and training conditions. Fluency was marginally greater in adolescents trained in rule-switching vs. those trained in general ideation. Adolescents the rule-switch training condition had greater flexibility scores than the adolescents in the creative and general ideation conditions. In adults, the opposite was observed for flexibility, where adults trained in creative ideation outperformed the active control groups in flexibility. For unicity, adolescents had marginally lower scores indicating greater uniqueness of solutions. ^*^ *p* < 0.05.

***Alternative Uses Tin Can and Brick***. The AU Brick task was the posttest counterpart of the AU Tin Can pretest task. Originality, fluency, flexibility, and unicity (inverse of uniqueness) were measured on the AUT. Results are shown in Figure 4.

No main effects for Condition or Age were found for originality [Condition: *F*_(2, 104)_ = 0.10, *p* = 0.91, η^2^_*p*_ = 0.00, Age: *F*_(2, 104)_ = 1.48, *p* = 0.23, η^2^_*p*_ = 0.01]. Also, no Age × Training Condition interaction was found on the measure of originality [*F*_(2, 104)_ = 1.01, *p* = 0.37, η^2^_*p*_ = 0.02].

For fluency there were no main effects for Condition [*F*_(2, 104)_ = 1.44, *p* = 0.24, η^2^_*p*_ = 0.03] or Age [*F*_(2, 104)_ = 3.33, *p* = 0.07, η^2^_*p*_ = 0.03]. There was a significant interaction effect between Age and Condition on fluency [*F*_(2, 104)_ = 3.16, *p* = 0.047, η^2^_*p*_ = 0.06]. Therefore, an additional ANCOVA per age-group with Bonferroni-corrected *post-hoc* comparisons was conducted. These analyses revealed a marginally greater fluency in adolescents in the rule-switching condition vs. the general ideation condition (Δ*M* = 5.62, *SE* = 2.31, *p* = 0.05). No other significant differences between the training conditions were found.

For flexibility there were also no main effects for Condition [*F*_(2, 104)_ = 1.19, *p* = 0.31, η^2^_*p*_ = 0.02], or Age [*F*_(2, 104)_ = 1.33, *p* = 0.25, η^2^_*p*_ = 0.01], The Condition × Age effect was significant: *F*_(2, 104)_ = 6.42, *p* < 0.01, η^2^_*p*_ = 0.11. This was investigated further with an ANCOVA per age-group with Bonferroni corrected *post-hoc* tests for Condition. These revealed greater flexibility for the Rule-switching than General ideation training condition in adolescents (Δ*M* = 2.18, *SE* = 0.74, *p* = 0.01) and marginally greater flexibility for the Creative ideation vs. Rule-switching condition in adults (Δ*M* = 2.09, *SE* = 0.84, *p* = 0.05). No other significant differences between training conditions were found.

Adolescents had marginally lower scores for unicity (i.e., higher scores infer less unique solutions) compared to adults [*F*_(1, 104)_ = 3.82, *p* = 0.05, η^2^_*p*_ = 0.03], indicating greater uniqueness of solutions for adolescents. There was no main effect for Condition [*F*_(2, 104)_ = 0.25, *p* = 0.78, η^2^_*p*_ = 0.00], nor was there an interaction effect for Condition × Age [*F*_(2, 104)_ = 0.98, *p* = 0.38, η^2^_*p*_ = 0.02].

AU Tin Can performance was positively related to AU Brick performance; although it was not a significant covariate for originality [*F*_(2, 104)_ = 2.58, *p* = 0.11, η^2^_*p*_ = 0.02], it did form a significant covariate for fluency [*F*_(2, 104)_ = 5.78, *p* = 0.02, η^2^_*p*_ = 0.05], flexibility [*F*_(2, 104)_ = 8.78, *p* < 0.01, η^2^_*p*_ = 0.08], and unicity [*F*_(2, 104)_ = 8.04, *p* = 0.01, η^2^_*p*_ = 0.07]. In general this shows that individuals with high pretest “Tin Can” scores also obtained high posttest “Brick” scores.

#### General ideation

**Figure 5:**
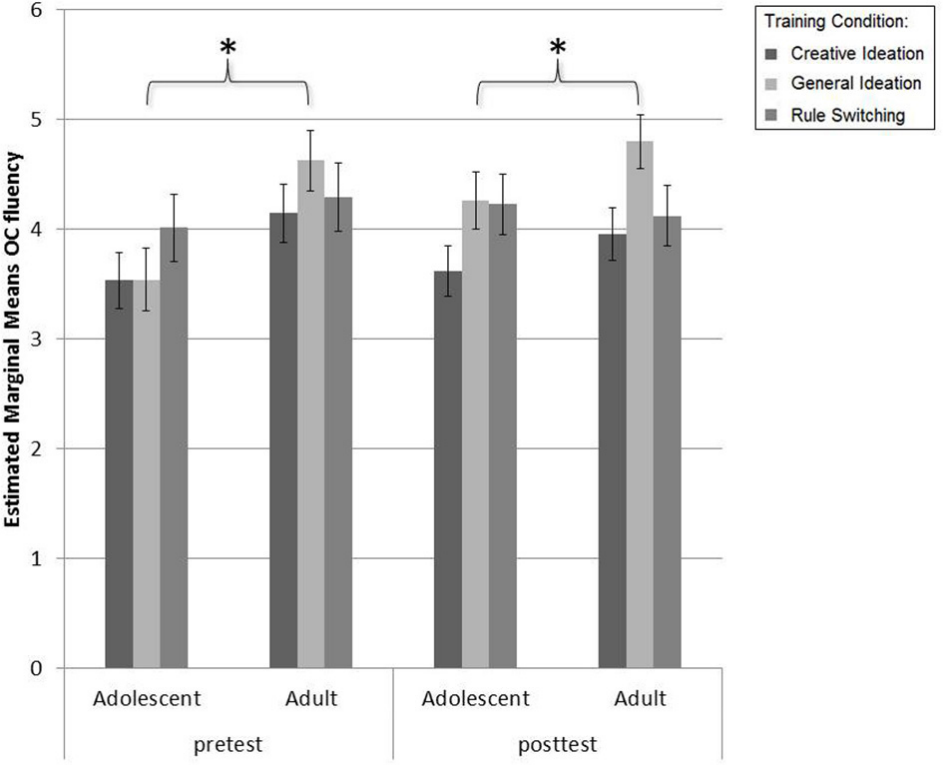
**Pretest to posttest progression for adults and adolescents on the general ideation measure of the combined Alternative Uses/Ordinary Characteristics task**. Adults had higher mean OC fluency scores (number of ordinary characteristics listed) on average; however, no other main or interaction effects for session, age group or training condition were found in OC fluency performance. ^*^ *p* < 0.05.

Repeated measures ANOVAs for OC fluency revealed no significant changes across sessions [Session effect: *F*_(1, 98)_ = 1.69, *p* = 0.20, η^2^_*p*_ = 0.02]. There was a main effect of Age [*F*_(1, 102)_ = 5.71, *p* = 0.02, η^2^_*p*_ = 0.05; see Figure 5] where adults obtained higher OC fluency scores compared to adolescents. No significant differences in OC fluency progression were observed for the two age groups [Session × Age effect: *F*_(2, 102)_ = 3.54, *p* = 0.06, η^2^_*p*_ = 0.03] or training conditions [Session × Condition effect: *F*_(2, 102)_ = 2.20, *p* = 0.12, η^2^_*p*_ = 0.04] or Age × Condition [Session × Age × Condition effect: *F*_(2, 102)_ = 0.15, *p* = 0.87, η^2^_*p*_ = 0.00].

#### Rule-switching

Performance on the RS task comprised measures of switch costs (mean repeat trial minus mean switch trial) for accuracy and reaction time. Participants trained with the RS task were expected to improve more than those trained in AU or OC.

**Figure 6:**
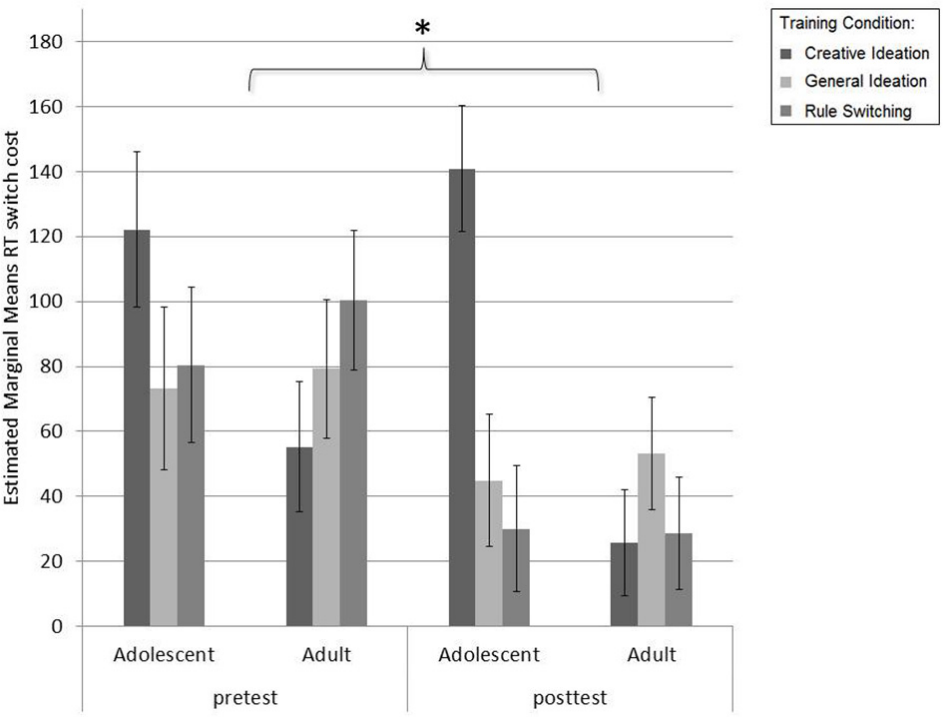
**Reaction time switch costs (ms) from pretest to posttest for adults and adolescents per training condition**. Switch costs were significantly lower on posttest than pretest. Adolescents decreased marginally more in switch costs than adults. Individuals trained in rule-switching decreased marginally more than those trained in creative ideation or general ideation. *Post-hoc* comparisons of a significant Age × Condition effect did not reveal further differences. ^*^ *p* < 0.05.

Switch costs decreased for accuracy from pretest to posttest [Session effect: *F*_(1, 76)_ = 5.36, *p* = 0.02, η^2^_*p*_ = 0.07]. A Session × Age interaction was found for accuracy [*F*_(1, 76)_ = 9.40, *p* < 0.01, η^2^_*p*_ = 0.11], where adolescents decreased more in switch costs than adults (see Figure 6). No Session × Condition or Session × Condition × Age effects were found for accuracy [*F*_(1, 76)_ = 0.61, *p* = 0.55, η^2^_*p*_ = 0.02. or *F*_(1, 76)_ = 0.07, *p* = 0.93, η^2^_*p*_ = 0.00]. There were no main effects for Age [*F*_(1, 76)_ = 0.02, *p* = 0.89, η^2^_*p*_ = 0.00] or Condition [*F*_(1, 76)_ = 0.59, *p* = 0.56, η^2^_*p*_ = 0.02] or Age × Condition [*F*_(1, 76)_ = 1.29, *p* = 0.28, η^2^_*p*_ = 0.03].

For reaction time, switch costs also decreased from pretest to posttest [Session effect: *F*_(1, 76)_ = 10.97, *p* < 0.01, η^2^_*p*_ = 0.13]. No Session × Age or Session × Age × Condition interactions were present [*F*_(1, 76)_ = 1.42, *p* = 0.24, η^2^_*p*_ = 0.02 and *F*_(1, 76)_ = 0.60, *p* = 0.55, η^2^_*p*_ = 0.02, respectively]. No main effects for Age [*F*_(1, 76)_ = 3.16, *p* = 0.09, η^2^_*p*_ = 0.04] or Condition [*F*_(1, 76)_ = 1.45, *p* = 0.24, η^2^_*p*_ = 0.04] were present. A marginal Session × Condition interaction effect was present [*F*_(1, 76)_ = 3.01, *p* = 0.06, η^2^_*p*_ = 0.07] and a significant Age × Condition interaction was present for reaction time [*F*_(2, 76)_ = 5.76, *p* < 0.01, η^2^_*p*_ = 0.13]. Follow-up repeated measures analyses for reaction time were conducted per age group in order to further investigate the role of training condition. These *post-hoc* comparisons with Bonferroni correction revealed no significant differences within age groups between training conditions.

### Progression during training

We used repeated measures ANOVAs with Age (adolescent, adult) as between-subjects factor and Session (1–8) as within-subjects factor to examine the participants' progression during training. Homogeneity of variance between factors was examined with Levene's test. Greenhouse-Geisser correction for any violations of sphericity was applied when required. In some cases training data for one session was incomplete due to loss of Internet connection or early closing of the training software Internet browser (N_AU_ = 6, N_OC_ = 11, N_RS_ = 10); when this occurred the session score was computed based on the mean of the previous and next session. Participants for whom data from more than one consecutive session was incomplete were excluded from the analyses (N_AU_ = 2, N_OC_ = 2, N_RS_ = 2).

#### Creative ideation training

**Figure 7:**
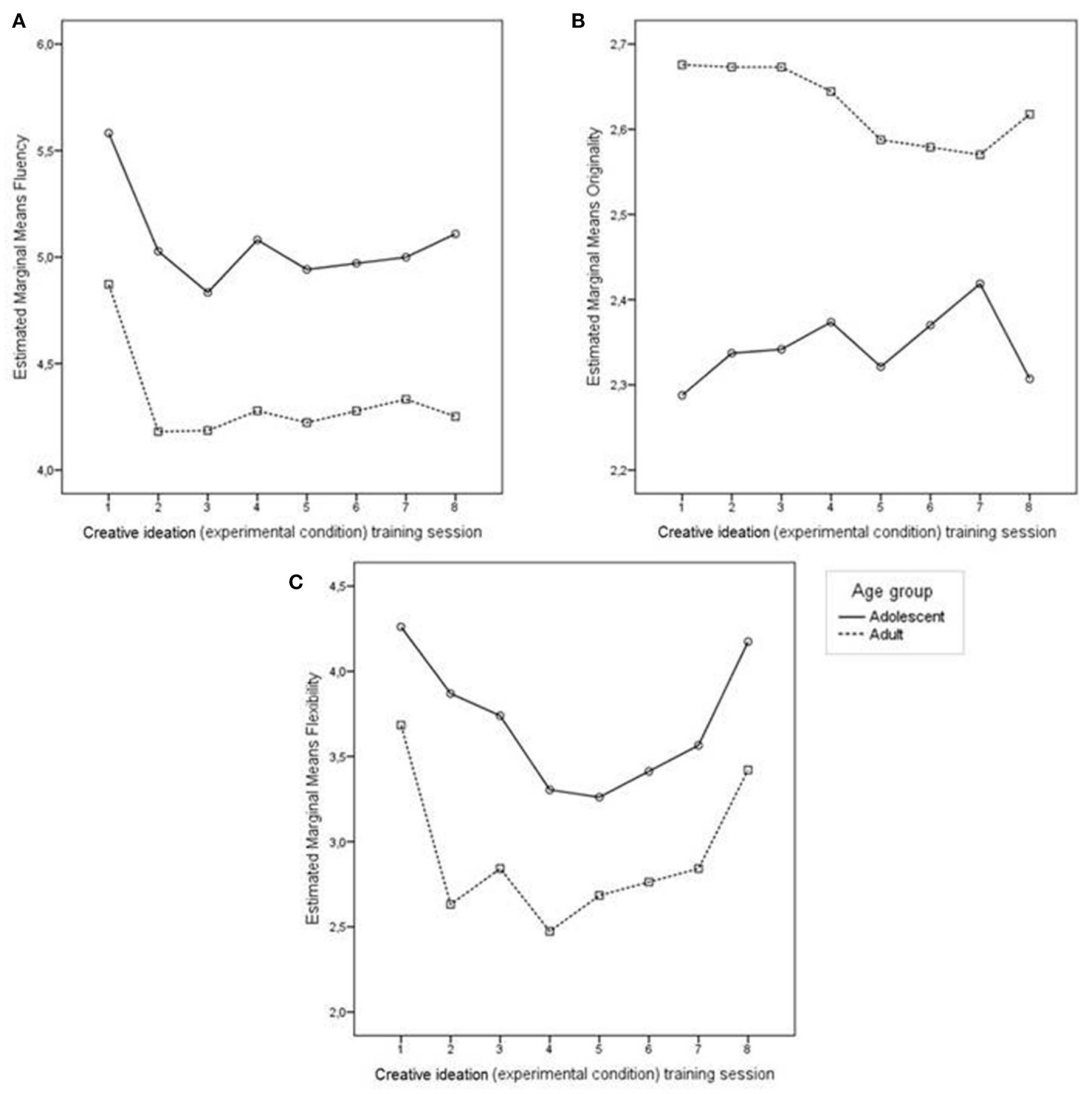
**Progression during Alternative Uses (experimental) training across sessions for adults and adolescents: (A) fluency (number of solutions), (B) originality (1 = “not original” to 5 = “highly original”), (C) flexibility (number of categories used in solutions)**. There were no significant age group differences in fluency. Adults scored higher on originality throughout the training sessions. Adolescents displayed greater flexibility during the course of the training.

A depiction of the participant's progression on the measures of originality and fluency, flexibility on the Alternative Uses (AU) training task is shown in Figure 7. Adults on average had higher scores on the originality measure [*F*_(1, 44)_ = 9.01, *p* < 0.01, η^2^_*p*_ = 0.17], whereas as adolescents on average had marginally higher scores for flexibility [*F*_(1, 44)_ = 3.93, *p* = 0.05, η^2^_*p*_ = 0.09]. There were no differences between age groups on the fluency measure [*F*_(1, 44)_ = 0.57, *p* = 0.46, η^2^_*p*_ = 0.01].

Although there was no main effect for Session on originality [*F*_(1, 44)_ = 0.12, *p* = 0.73, η^2^_*p*_ = 0.01], a significant quadratic Session effect emerged for flexibility [*F*_(1, 44)_ = 29.92, *p* < 0.001, η^2^_*p*_ = 0.42] and a significant cubic Session effect was present for fluency [*F*_(1, 44)_ = 5.55, *p* = 0.02, η^2^_*p*_ = 0.11]. Session × Age interactions were not present for originality [*F*_(1, 44)_ = 1.23, *p* = 0.30, η^2^_*p*_ = 0.42], fluency [*F*_(1, 44)_ = 0.18, *p* = 0.88, η^2^_*p*_ = 0.00] or flexibility [*F*_(1, 44)_ = 0.60, *p* = 0.65, η^2^_*p*_ = 0.01]. In short, results indicate that although training does not affect originality, it does impact both fluency and flexibility in creative ideation, two critical antecedents of original thinking and insight performance.

#### General ideation training

**Figure 8:**
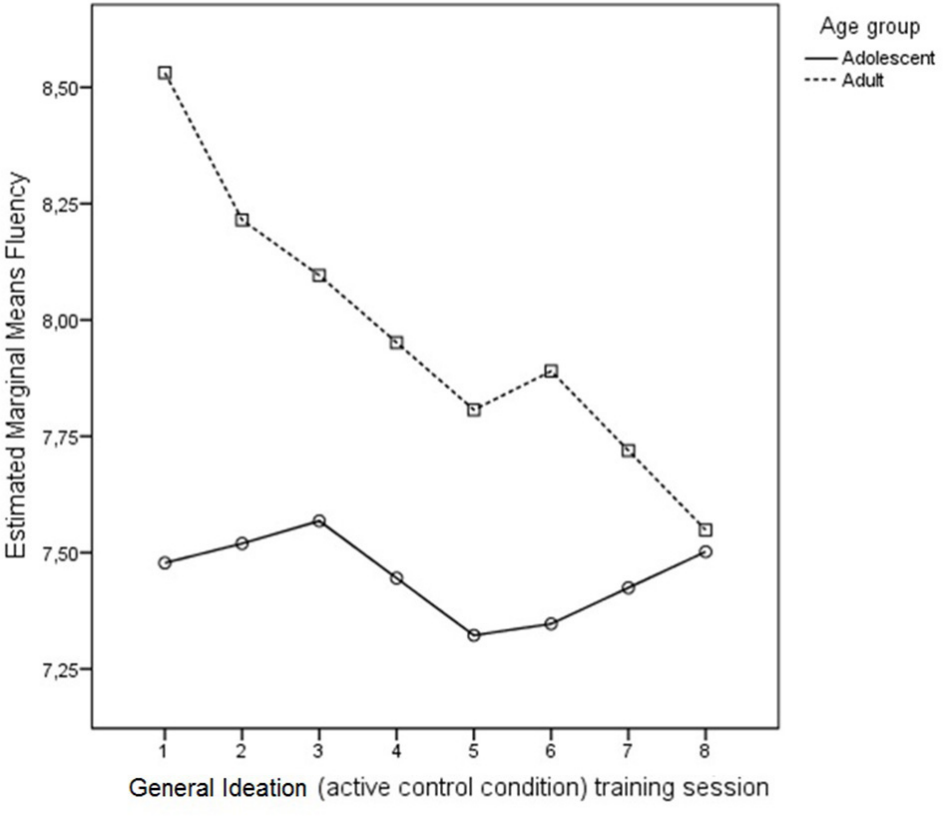
**Progression in number of solutions (fluency) during Ordinary Characteristics (active control) training across sessions for adults and adolescents**. No age differences in fluency of ordinary characteristics ideation were found.

Fluency performance for adults and adolescents on the Ordinary Characteristics (OC) training task is shown in Figure 8. Analyses do not show a main effect for Age [*F*_(1, 39)_ = 0.64, *p* = 0.46, η^2^_*p*_ = 0.01] nor a Session × Age interaction [*F*_(1, 39)_ = 0.96, *p* = 0.54, η^2^_*p*_ = 0.02]. Thus, no discernible differences were present in adolescents and adults progression on the OC task during the training sessions. Training does not affect general ideation.

#### Rule-switch training

**Figure 9:**
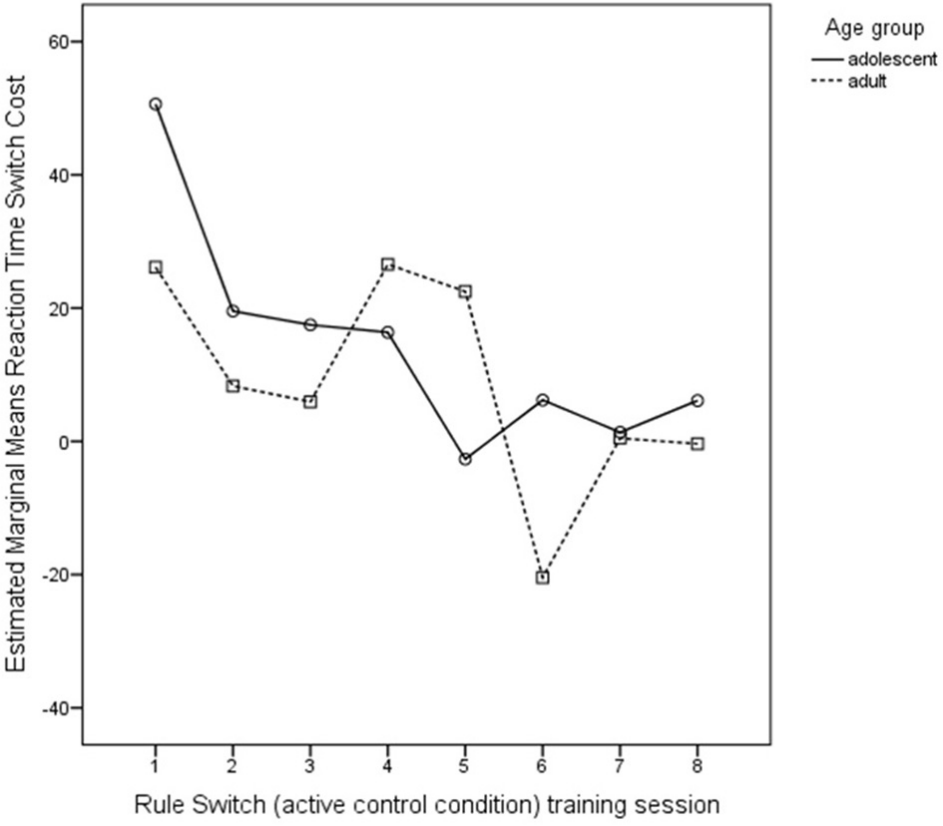
**Progression in reaction time switch costs (ms) during Rule-Switch (active control) training across sessions for adults and adolescents**. No age differences in reaction time switch costs were found.

Switch costs remained relatively steady across Sessions for both accuracy and reaction time [accuracy: *F*_(1, 39)_ = 1.73, *p* = 0.10, η^2^_*p*_ = 0.04; reaction time: *F*_(1, 39)_ = 1.67, *p* = 0.19, η^2^_*p*_ = 0.04], as can be seen in Figure 9. Adults and adolescents did not differ in average switch costs during training [accuracy: *F*_(1, 39)_ = 1.35, *p* = 0.25, η^2^_*p*_ = 0.03; reaction time: *F*_(1, 39)_ = 0.51, *p* = 0.48, η^2^_*p*_ = 0.01] throughout the training sessions. No interaction between Session and Age is present for accuracy [*F*_(1, 39)_ = 0.67, *p* = 0.66, η^2^_*p*_ = 0.02] or reaction time [*F*_(1, 39)_ = 0.64, *p* = 0.54, η^2^_*p*_ = 0.02]. As for training creative ideation, training does affect rule-switching ability yet not differently for age groups.

## Discussion

The aim of the current study was to examine the effects of creative ideation training in adolescents and adults. To this end, participants followed one of three training types; alternative uses generation (creative ideation condition), general ideation, or rule-switching. A set of tasks measuring both creative ideation and general cognitive functions were administered before and after 2 weeks of training. There were two main findings: (1) participants improved in creative ideation and rule-switching, and (2) adolescents benefitted more from training than adults, although this was independent of the type of training provided. The results are organized along these findings.

### Initial developmental differences

Before interpreting the effects of training, it is important to consider potential age differences prior to training. The prediction was that adults and adolescents would perform equally well on most creativity measures, but that adults would outperform the adolescent group on originality (Wu et al., 2005; Kleibeuker et al., 2013a). We anticipated additional differences for general ideation with better performance for adults compared to adolescents, based on prior research (Kleibeuker et al., 2013b) and its close relation to verbal fluency performance (Romine and Reynolds, 2005). No initial differences were expected for RS performance (Huizinga et al., 2006).

Results for creative ideation in the 4-min AUT resembled previous findings in which adolescents performed at a mature level on most aspects of creativity, including fluency and flexibility. Also fitting earlier work, significant developmental differences were apparent on the measure of originality, with more original and unique solutions for adults compared to adolescents (see also Kleibeuker et al., 2013b). Different factors may account for these developmental differences. First, given their greater knowledgebase and more lifetime experience (e.g., Weisberg, 1999), adults have a greater chance of retrieving original and unique associations with presented objects. Also, individual lifestyles of adults generally involve larger inter-individual variance in experiences in comparison to adolescents. Consequently, adults are more likely to create relatively infrequent and unique associations and ideas. A second possible explanation for differences between age groups concerns developmental changes in flexible coordination between analytic and associative processing (Martindale and Hasenfus, 1978; Martindale, 1999; Christoff et al., 2009a,b), which is associated with functioning of prefrontal brain regions that develop throughout adolescence and into adulthood (Kerns et al., 2004; Kerns, 2006). Both analytic and associative processing are believed to lead to numerous creative ideas (Nijstad et al., 2010; De Dreu et al., 2012; Kleibeuker et al., 2013c); however, the quality of generated ideas has been related to the ability to flexibly coordinate between analytic and associative processing. Thus, adolescent participants may not yet have fully developed the ability to successfully shift between the two types of processing (Smolucha and Smolucha, 1986; see also Runco, 1991).

As predicted, we found developmental differences in fluency on general ideation. As with age related differences in creative ideation, and originality in particular, this effect could be explained by age related differences in experiences and knowledge base. A second explanation concerns the development of processes that are related to memory retrieval. These processes are associated with lateral prefrontal cortex activations (e.g., Buckner et al., 2008) and other brain regions that develop relatively late and mature throughout adolescence (Giedd et al., 1999; Fair et al., 2007). Consistent with prior studies no age related differences were observed for performance on the RS task (Huizinga et al., 2006), suggesting that cognitive flexibility is already at adult level in middle adolescence.

### Training effects

The applied training paradigm revealed several interesting findings. Participants improved in creative ideation and rule-switching. More specifically, the RS training group improved on the RS task, with larger performance increases relative to the other two training groups (e.g., Karbach and Kray, 2009). Training effects were also observed for creative ideation; however, contrary to what was observed for the RS training, these benefits were not specific to the creative ideation group. There were general increases for all training conditions on originality and fluency on the multiple object AUT. No improvements were observed for general ideation.

The general improvement in fluency and originality could be interpreted as follows. First, given that these effects were non-specific for training conditions, it is possible that the improvements for all three conditions, including the creative ideation training, simply reflect retesting effects instead of training effects. Indeed, some crucial aspects of the creative ideation task differed from the training paradigm such as duration (20 s vs. 2 min), way of answering (audio recording, typing), and task switches (alternative uses to ordinary characteristics vs. only one task during training). The task might therefore test processes that are different from those applied during the creative ideation training sessions. However, the correlations between the alternative uses training task and the two AUTs administered during pretest and posttest suggest that the improvements for the alternative uses training group are at least to some degree related to their practice with the AUT. Perhaps simply practicing with the AUT was not enough to elicit a discernible effect and more extensive training informing people about the nature of creativity and strategies for creative thinking (e.g., Speedie et al., 1971; Clapham, 1997; Scott et al., 2004) or providing exposure to ideas of others (Dugosh and Paulus, 2005; Fink et al., 2010) would improve the impact of creative ideation training. This hypothesis can be studied in future research by examining the effect of different types of training programs with AUTs of varying lengths. A second explanation may be that practice generating ordinary characteristics or with the rule-switching task may benefit generating alternative uses (performance) through improvements of processes that support creative ideation. Improvements in cognitive flexibility as practiced in the rule-switching condition may benefit generating alternative uses as well as switching between tasks during the combined alternative uses and ordinary characteristics task. Indeed, cognitive flexibility is thought to be important for creative performance (e.g., Warren and Davis, 1969; Gilhooly et al., 2007; Baas et al., 2008; De Dreu et al., 2008; Nusbaum and Silvia, 2011; Bott et al., 2014). Furthermore, originality and fluency in the generation of alternative uses could be enhanced by improving the ability to successively retrieve relevant semantic information from memory, i.e., general fluency as was the case during the ordinary characteristics task training. For example, creativity training in which participants were instructed to retrieve information about the parts that make up the object appeared to be effective (Warren and Davis, 1969). This role of our two active control tasks can be examined by administering the alternative uses and ordinary characteristics tasks separately.

### Developmental differences in training effects

An important question in this study concerned whether training benefits would be larger for adolescents than adults. Interestingly, greater increases in originality and uniqueness were observed for adolescents compared to adults independent of training condition. These findings suggest that adolescence is a period of enhanced susceptibility for training effects. Indeed, prior research on cognitive training indicates that at least for certain higher cognitive functions, adolescents have greater potential for improvement than adults (Jolles and Crone, 2012). These developmental differences can be attributed to developmental changes in brain structure and function. Increasing specialization and integration of brain regions with age are argued to result in decreased plasticity of cognitive functions in adults compared to adolescents (Huttenlocher, 2003; Johnson, 2011; see also Jolles and Crone, 2012). Moreover, adolescence is a period associated with the reorganization of the prefrontal cortex and related regulatory systems (Keating, 2004; Steinberg, 2005). Given the strong associations between creative ideation, prefrontal cortex and cognitive control functionality (e.g., Groborz and Necka, 2003; Dietrich, 2004; Keating, 2004), adolescence provides a favorable time window for progression in creative ideation.

Another explanation concerns developmental differences in flexibility in learning. Recent rodent studies indicate that (young) adolescents, in comparison to adults, learn more flexibly; they are less prone to training induced perseverance and show greater flexibility in reversing learned associations (Johnson and Wilbrecht, 2011). Indeed, generating original ideas, especially through the *flexibility pathway*, is associated with flexible switching between (distant) associations and overcoming perseverance of cognitive biases or “functional fixedness” (Baas et al., 2008; Nijstad et al., 2010). This latter explanation particularly concerns training effects within the same domain, but also likely operates on associations formed during practice with the ordinary characteristics task. According to the flexibility hypothesis, adolescents would not or at least be less susceptible to training induced automaticity and perseverance.

A second age related finding concerns different effects of training paradigm for adults and adolescents on divergent thinking fluency and flexibility. More specifically, the current results indicate that task switch training in adolescents has a larger effect on creative ideation flexibility than in adults. These results suggest that adolescents and adults employ different processes or strategies to generate alternative uses, with more reliance on cognitive flexibility functions for the adolescent age group. Thereby, these findings provide further support for the hypothesis that adolescence is a developmental stage of increased flexibility optimized for adaptive and explorative behavior during this life phase of instability (Johnson and Wilbrecht, 2011; Crone and Dahl, 2012).

### Limitations

Some limitations of this study deserve mention and can be informative for future research. First, the absence of a control group without training made it difficult to distinguish between re-test effects and training effects as well as examine the existence of transfer effects to posttests. Future studies should therefore incorporate a passive control group. Second, task choices may have obscured some of the training effects. The single object AUT (Tin Can and Brick) differed in difficulty and coding scheme and could not be directly compared to examine pretest to posttest change. Future studies would most likely benefit from implementing a multiple object assessment at each time point, which may represent a purer measure of creative ideation as individual differences in the necessary knowledge of the different objects is spread out thus reducing measurement error. Third, the current study does not provide information about long-term effects of the training. Retesting after, for example, a 6 month period would provide additional information on the effects of the different training paradigms and plasticity in adolescents, which might be especially informative for educational purposes. Fourth, the results were not controlled for motivation differences. Adolescence has been argued to be a developmental stage where motivation effects are more prominent than adulthood (Steinberg, 2005); therefore, incorporation of motivation questionnaires might provide insight into possible side effects of individual differences in motivation. Finally, this study focuses only on creative ideation in the verbal domain; in future studies other domains such as figural divergent thinking or visual insight should be investigated.

## Conclusions and future directions

In future research, it would be interesting to gain better understanding of the observed developmental differences in training effects also reflect underlying changes. It would be of particular interest to test whether the observed changes in creative thinking performance for the different types of training (alternative uses generation, ordinary characteristics retrieval, and rule-switching) are the consequence of changes in similar or perhaps different underlying functions. As such, future research could focus on training-related neuronal changes using (f)MRI, especially in prefrontal regions, known to be related to creative thinking (Keating, 2004). Moreover, it would be interesting to focus on age related effectiveness of different training paradigms. In the current study, 13–15 year olds were compared to 22–30 year olds. Testing a larger range of ages, including pre-adolescents and late adolescents, would provide a more detailed perspective of development-related limitations and opportunities in training of creative ideation. For the current study, our aim was to better understand the effects of practice only in adults and adolescents. An interesting addition could be informing people about the nature of creativity and strategies for creative thinking, or use an adaptive design, distinguishing between levels of task difficulty, both of which have been shown to be effective interventions (e.g., Speedie et al., 1971; Clapham, 1997), but knowledge about developmental differences in effectiveness is still lacking. Interestingly, the amount of feedback provided by the trainer had a substantial negative impact on the divergent thinking training effectiveness in earlier studies (Scott et al., 2004). However, peer feedback in the form of idea sharing (Paulus and Nijstad, 2003) and exposure to ideas from others (Dugosh and Paulus, 2005; Fink et al., 2010) does appear to enhance creativity. Adolescents react differently to feedback from peers than adults (Albert et al., 2013), thus an investigation into developmental differences in the effect of peer feedback could be another interesting addition to the creativity training literature.

The results of the current study not only contribute to the fundamental knowledge of cognitive development, but also provide possible implications with regard to creativity education and training. Indeed, the present results imply that adolescence is an advantageous period to enhance “out of the box” thinking and creative processes. Given the importance of creative thinking to individual life success and societal improvement (e.g., Ward et al., 1999), educators should take advantage of this sensitive period to improve divergent thinking skills.

In conclusion, the results support earlier findings in showing that practice in creative ideation is successful within the same domain (Scott et al., 2004) and supports the hypothesis that adolescence is a developmental stage of increased flexibility optimized for adaptive and explorative behavior during this instable life stage (Johnson and Wilbrecht, 2011; Crone and Dahl, 2012).

### Conflict of interest statement

The authors declare that the research was conducted in the absence of any commercial or financial relationships that could be construed as a potential conflict of interest.
